# Opportunities and challenges harnessing antigen-specific CD4+ regulatory T cells in inflammatory bowel disease

**DOI:** 10.3389/fimmu.2025.1667053

**Published:** 2025-10-29

**Authors:** Jessica Kümmel, Nicolas Schlegel, Johanna C. Wagner

**Affiliations:** Department of General, Visceral, Transplantation, Vascular and Pediatric Surgery, University Hospital Würzburg, Würzburg, Germany

**Keywords:** regulatory T cell, CAR receptor, inflammatory bowel diseases, flagellin- specific CAR, IL23R-CAR, cell therapy, immunotherapy

## Abstract

Inflammatory bowel diseases (IBD), including Crohn´s disease (CD) and ulcerative colitis (UC) remain highly prevalent and are associated with a reduced quality of life in affected patients. The pathophysiology of IBD is multifactorial since genetic predisposition, altered immune function, changes in intestinal microbiota, environmental factors, and loss of intestinal barrier function together induce disease manifestation. A critical key factor is the dysregulation of the immune system which explains that all medical therapeutic approaches target the immune response. However, the success of these therapies is limited and associated with severe side effects which demonstrates the need for novel therapeutic approaches. Previous research demonstrated that CD4+ regulatory T (Treg) cells are important regulators of intestinal homeostasis but are reduced in number and function relative to effector T cells in IBD. This led to the concept that genetically engineered, antigen-specific Tregs may represent a promising strategy to address immune dysregulation in IBD. Due to their antigen specificity, chimeric antigen receptors (CARs) enable additional target-dependent activation and migration of Tregs at disease sites. While CARs are increasingly established for the generation of antigen-specificity for T cell therapies in cancer, the implementation of CARs for IBD is in a preliminary state. Nonetheless, CAR constructs specific to circulating carcinoembryonic antigen (CEA), flagellin, or IL23R have been developed recently for potential application in IBD. Based on these novel developments, this review will discuss the role of Tregs in IBD disorders and present the current state of CAR Treg models.

## 1 Introduction

Crohn’s disease (CD) and ulcerative colitis (UC) are the major types of inflammatory bowel disease (IBD). The prevalence and incidence of these inflammatory disorders have risen over the past decade (1, 2). Currently, a stable, yet high incidence level was observed in countries such as North America and Europe, while the incidence increased significantly in newly industrialized countries, including Africa, Asia, and South American countries, such as Brazil (1).

CD is an inflammatory disease that involves all layers of the intestinal wall and can affect any part of the gastrointestinal tract. It is characterized by episodes of remission and exacerbation (3). CD can have extra-intestinal manifestations, such as oral, musculoskeletal, dermatological, or ocular manifestations (4, 5). Both sexes are equally affected and the median age of onset is 30 years, with two peaks of onset between 15 and 30 years and around 50 years. Symptoms include diarrhea, abdominal pain, weight loss, and nausea. Oral manifestation symptoms contain diffuse lip or mouth swelling, esophageal lesions redness and edema (4, 6). UC is often diagnosed in late adolescence and early adulthood affecting both sexes equally (7). Inflammation is typically restricted to the mucosa, begins in the rectum and may extend continuously through the colon with variable extent or involve the entire mucosal surface (7, 8). Over time, there is often an alternation between periods of progression and regression of the inflammation (7). Symptoms include rectal bleeding, bloody diarrhea, rectal urgency, anemia, weight loss, fever, tachycardia, tenesmus, or nausea (7, 9).

The pathogenesis of IBD is multifactorial as genetic factors, such as heredity or mutations and non-coding variations, as well as environmental factors, including diet, microbiota, and medication influence the development of disease (10, 11). Another key factor is the dysregulation of the immune system, including the initiation of inflammation by triggering a pro- and anti-inflammatory cascade. Immune cells, such as B cells, T cells, macrophages, dendritic cells, and neutrophil granulocytes infiltrate the effector site where the immune response is dysregulated. The infiltrating cells secrete cytokines, such as TNF, interferon-gamma (IFN-γ), interleukins, and other cytokines that activate pro-inflammatory pathways (11, 12). The loss of Treg number and function in the gut wall significantly contributes to the onset of inflammation in IBD (13–15). Current medical treatments for IBD are limited in their efficacy (16), so novel therapeutic approaches are necessary.

Consequently, Tregs have emerged as a promising target for therapeutic interventions in IBD. Thus, in order to achieve disease specificity and re-direct Tregs to the site of inflammation, Tregs with a genetically engineered chimeric antigen receptor (CAR) have been developed (17). Up to now, a few promising pre-clinical studies in IBD models have produced encouraging results, paving the way for subsequent clinical studies. Those already developed CAR Treg models include CEA-specific, Flagellin-specific, and IL23R-CAR Tregs. Prior to clinical translation, it is imperative to address safety concerns, limitations in availability, prevention of CAR Treg exhaustion, and enhancements in phenotypic stability and suppressive function (17, 18).

The following review will give an overview of Tregs and the current state of research on Tregs in IBD as well as the various engineered CAR Treg models that have been developed for the treatment of IBD. With this we highlight the emerging potential of CAR-engineered Tregs as a novel therapeutic option.

## 2 Regulatory T cells in health and IBD disorders

### 2.1 Regulatory T cells as immune regulators

In the context of IBD, a dysregulation of pro-inflammatory and anti-inflammatory immune cells has been observed (19, 20). The transition to a pro-inflammatory milieu leads to the increased recruitment of pro-inflammatory immune cells, including neutrophils, macrophages, B cells, and T effector cells (21, 22). This results in a cytokine-rich environment, replete with pro-inflammatory cytokines, such as TNF-α, IL-1ß, and IL-6 (23). Therefore, in order to counteract this pro-inflammatory milieu, it is imperative to enhance the anti-inflammatory response. Therefore, immune cells with anti-inflammatory properties are pivotal actors in this process, such as regulatory T cells (24).

CD4+ regulatory T cells play a pivotal role in the mechanism of immune tolerance and comprise approximately 10% of the total CD4+ T cell population (25, 26). The defining characteristics of Tregs include the expression of cell surface receptors CD25 (IL-2 receptor-α chain) and cytotoxic T lymphocyte-associated antigen 4 (CTLA-4), along with low expression of CD127 (27–29). Another molecular marker is the Treg master lineage factor forkhead box protein P3 (FOXP3). This transcription factor is encoded by the X chromosome and is crucial for the development and function of Tregs (27, 28). The differentiation process of Treg cells is associated with the induction of FOXP3, for instance, through T cell receptor (TCR) signaling strength or by cytokines such as IL-2, IL-7, or IL-15, which act through the gamma chain of the cytokine receptor. This facilitates the induction of FOXP3 and, consequently, the differentiation process (30, 31). Co-stimulatory signals through CD28, particularly the Lck-binding domain of the CD28 cytoplasmic tail, appear to be involved in the induction of the transcription factor FOXP3 expression and the activation of Tregs (32).

**Figure 1:**
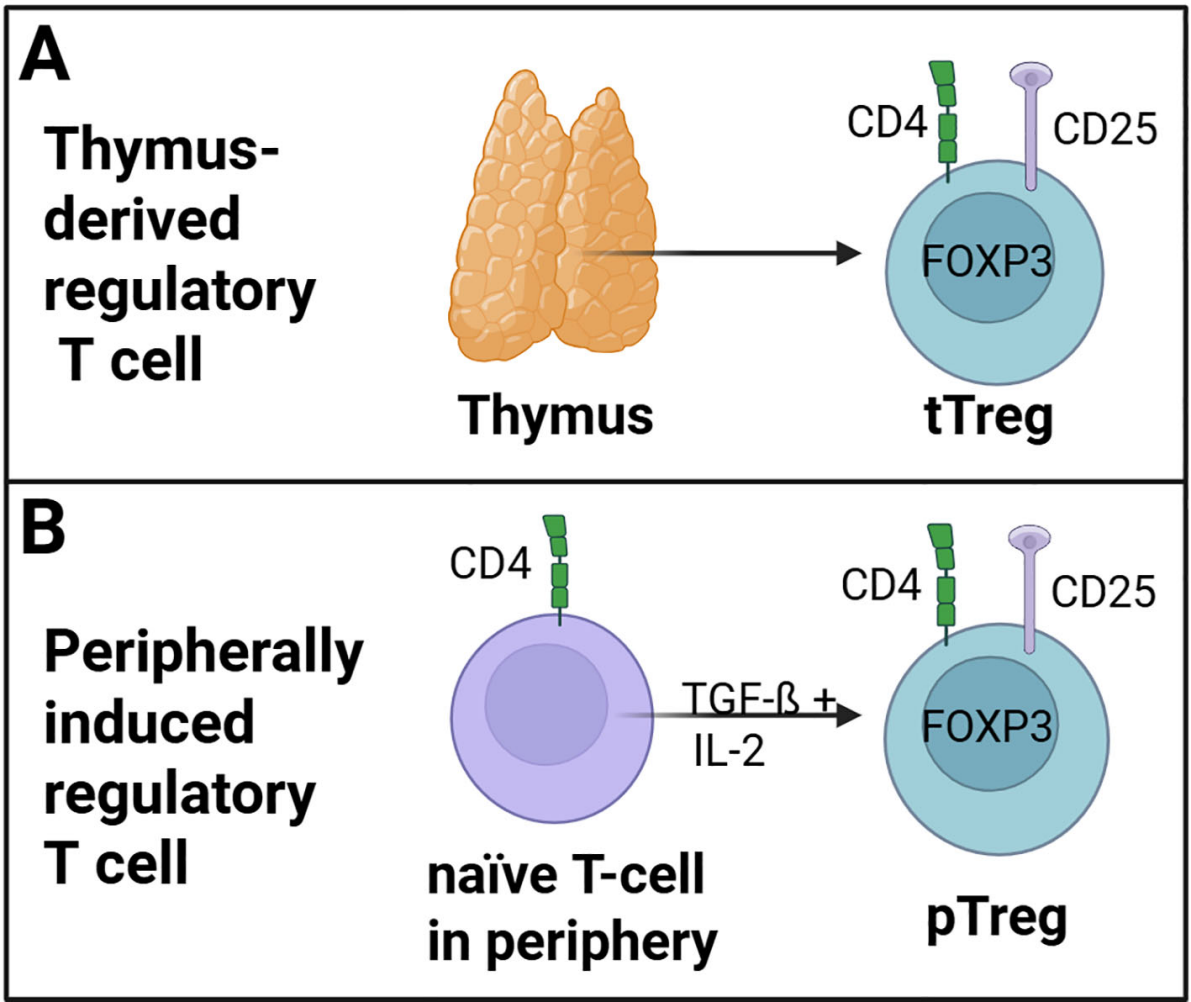
Regulatory T cell (Treg) subsets. **(A)** tTregs are derived from naïve CD4+ T cells within the thymus. **(B)** pTregs developed as naïve CD4+ T cells and transformed into FOXP3-expressing pTregs cells in the peripheral tissue trough TGF-ß and IL-2 stimulation. (Created in https://BioRender.com).

In general, the population of CD4+ regulatory T cells comprises two distinct subsets: tTregs and pTregs, as illustrated in **Figure 1**. Thymus-derived Tregs (tTregs) are derived from CD4+ T cells within the thymus (**Figure 1A**) (33). In contrast, peripheral Tregs (pTregs) develop as naïve CD4+ T cells and transform into FOXP3-expressing regulatory T cells in peripheral tissues upon TGF-ß and IL-2 stimulation (**Figure 1B**) (19, 34, 35). Furthermore, differences in the expression of neuropilin 1 (NRP1) and the member of the Ikaros transcription factor HELIOS have been observed among different Treg populations, with tTregs expressing these proteins constitutively (36, 37). Helios+ Tregs have been shown to exhibit heightened suppressive activity (38). The FOXP3 expression is enhanced by this transcription factor through binding on the FOXP3-promotor (39). In addition, Helios may be a marker for thymic-derived Tregs (40).

**Figure 2:**
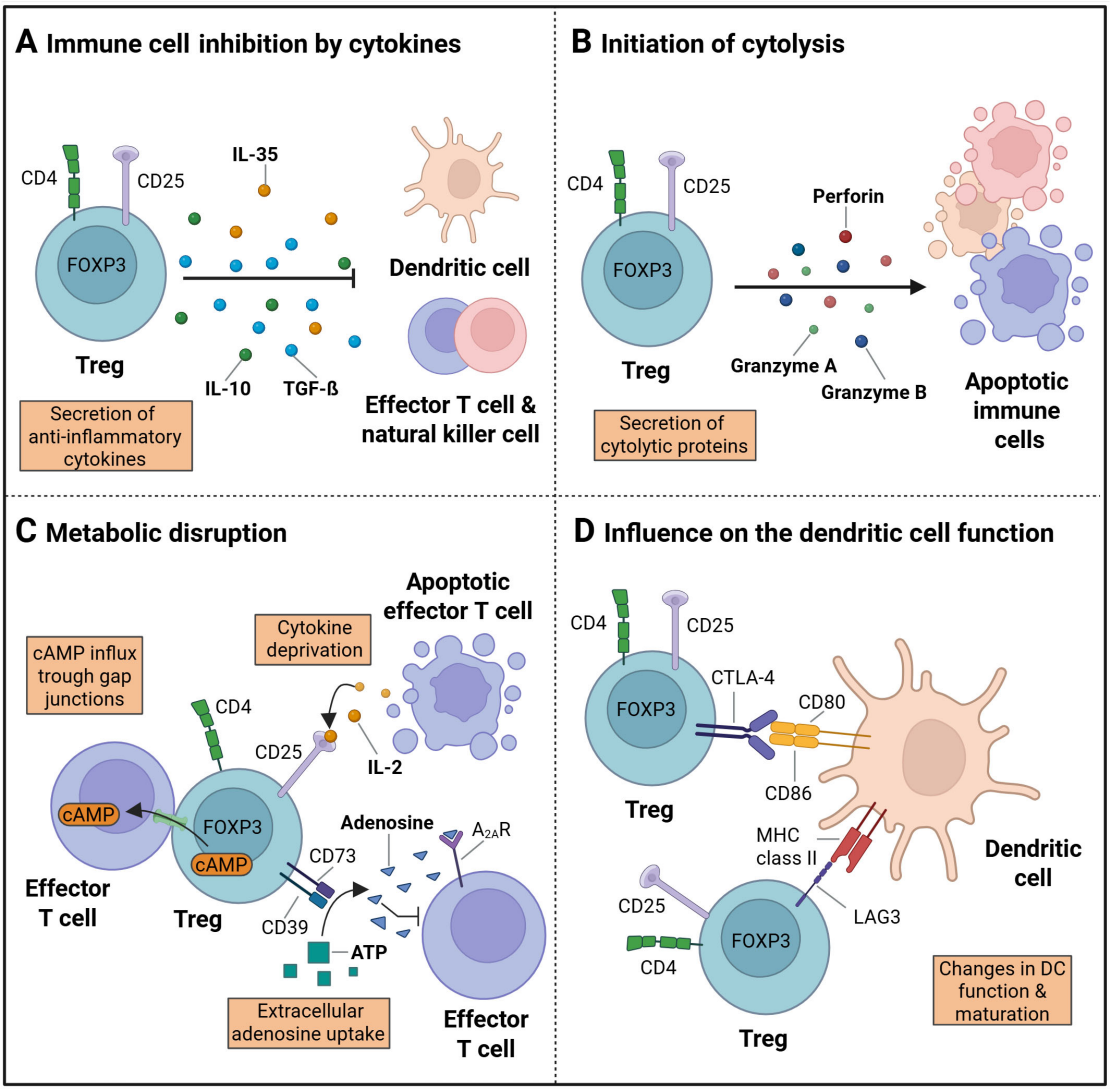
Treg immune regulation. **(A)** The process of immune suppression by regulatory T cells (Tregs) can be achieved through the secretion of cytokines, such as IL-10, IL-35 and TGF-ß. **(B)** The initiation of apoptosis following cytolysis is accompanied by the secretion of granzyme A and B, as well as perforins. **(C)** The disruption of metabolism is facilitated by the influx of cAMP into target cells via gap junctions, the deprivation of cytokines, and the production of extracellular adenosines. **(D)** The maturation and function of dendritic cells (DC) can be influenced by cell surface receptor proteins, including CTLA-4 and LAG3, which are expressed on Treg cells. (Created in https://BioRender.com). Adapted from “How regulatory T cells work” by Vignali, **(D)** et al, 2008, Nat Rev Immunol 8, 523-532.

The anti-inflammatory potential of Tregs is mediated through a variety of mechanisms, that are summarized in **Figure 2** (19, 41–43). One of these mechanisms involves the secretion of factors, such as inducible T cell co-stimulator (ICOS) and inhibitory cytokines, such as IL-10, IL-35, and TGF-ß (**Figure 2A**) (44–46). Furthermore, Tregs have been demonstrated to suppress the function of CD4+ effector T cells (Teffs) by blocking Teff protein synthesis through the secretion of IL-10 and TGF-ß (47).

Another mode of action is the suppression by cytolysis via secretion of granzyme A or B and perforin (**Figure 2B**). Granzymes A and B have been identified as critical mediators of cytolysis in effector T cells, NK cells, and CD8+ cytotoxic T cells (41, 48). Additional regulatory mechanisms encompass metabolic disruption (**Figure 2C**) (44). Metabolic disruption in the target cells is initiated through a variety of mechanisms, including cyclic AMP (cAMP)-mediated inhibition (49) or apoptosis caused by cytokine deprivation, specifically IL2 deprivation as Tregs exert a high IL-2 consumption (19). The presence of extracellular adenosine nucleosides has been observed to result in the suppression of effector T cell function, NK cell function, and DC maturation. Consequently, the adenosine A2A receptor (A2AR) is activated, and the process of extracellular adenosine formation from ATP by CD39 and CD73 is initiated (50, 51).

A further mechanism by which Treg cells modify the action of immune system cells involves the modulation of DC maturation and function (44). The suppression of DCs can be achieved via secreted cytokines (**Figure 2A**), initiation of cytolysis (**Figure 2B**), an uptake of extracellular adenosine nucleosides (**Figure 2C**), or the removal of co-stimulatory molecules (**Figure 2D**). The later has been shown to reduce the ability of DCs to stimulate effector T cells by binding of the CTLA-4 Treg surface receptor to CD80 and CD86 on the DC surface (52, 53). In addition, the maturation of DCs can be influenced and their function can be suppressed by binding of lymphocyte-activation gene 3 (LAG3) to MHC class II molecules on the DC surface (54–56).

### 2.2 Treg dysfunction in inflammatory bowel diseases

**Figure 3:**
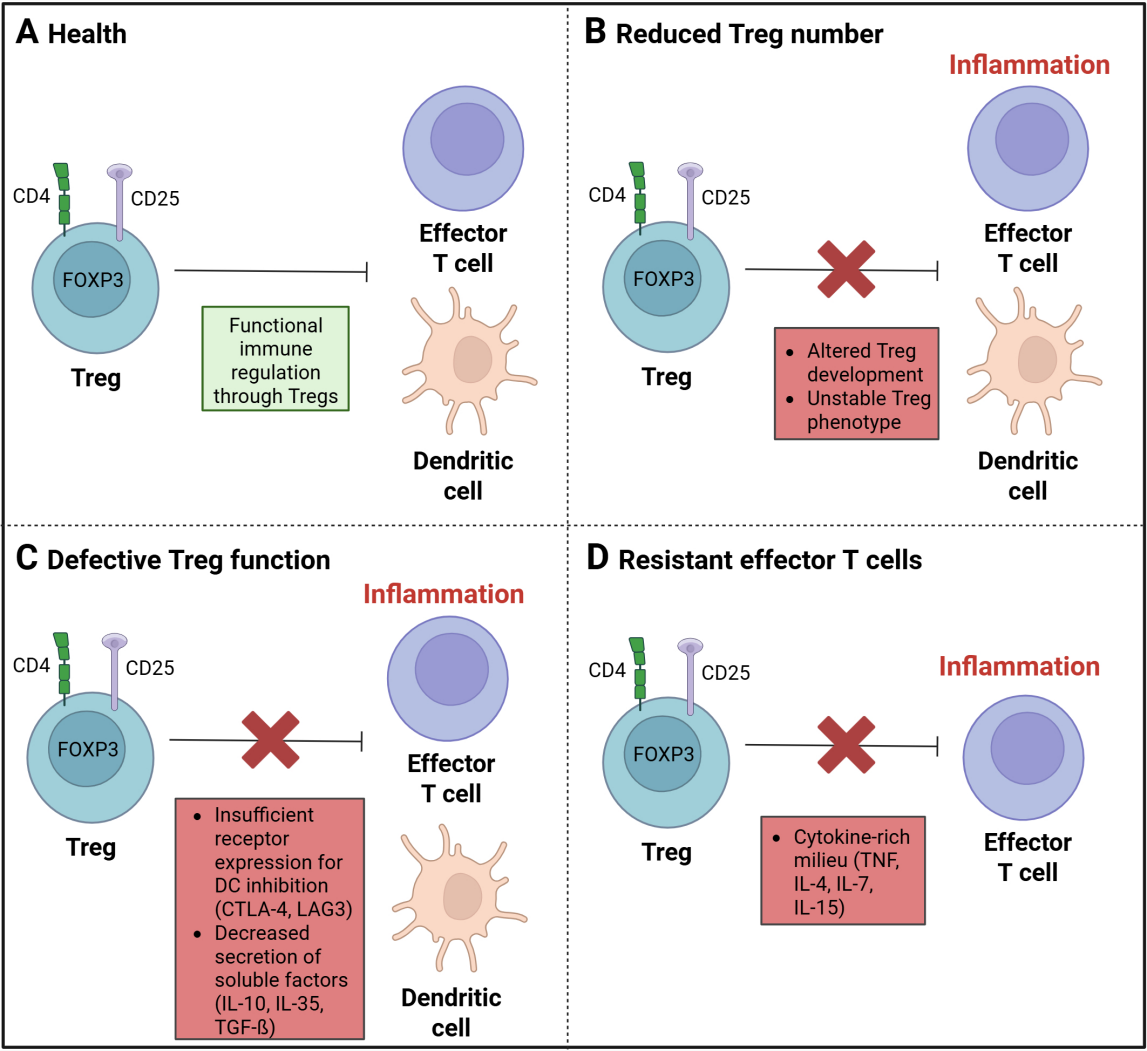
Treg dysregulation in inflammatory diseases. **(A)** A balanced and functional immune regulation by regulatory T cells (Tregs) is found in a state of health. **(B)** A disruption in immune regulation, resulting from a decrease in Treg cells, has been linked to a state of imbalance and dysfunction. Such a disturbance may be attributed to either an aberrant Treg development process or an unstable Treg phenotype. **(C)** The function of Treg cells can be affected by insufficient receptor expression for the DC inhibition through CTLA-4 or LAG3. A decrease in the secretion of soluble factors, such as IL-10, IL-35, or TGF-ß, may also result in dysfunction rather than significant anti-inflammatory effects. **(D)** A cytokine-rich milieu, comprising TNF, IL-4, IL-7, and IL-15, may improve the development of effector T cells (Teffs) and suppress the Treg function against Teffs. (Created in https://BioRender.com).

Tregs play a key role in the regulation of immune tolerance and in the control of inflammatory lesions in patients with IBD (**Figure 3A**). A defective function of Tregs in IBD can be a result of inherent defects and extrinsic and intrinsic alterations (57).

In some IBD patients, a reduced number of peripheral Tregs (20) (**Figure 3B**) as well as an increased level of soluble IL2Rα (CD25) in serum has been observed (58–60). Soluble IL2Rα has been identified as an inflammation marker, resulting from the proteolytic cleavage of the membrane-bound chain, a process known as shedding. This process contributes to the activation of T cells, as observed in the context of inflammation. Conventional CD4+ T cells have been found to produce a higher amount of soluble IL2Rα compared to Tregs (61, 62). The reduced number of regulatory T cells in active IBD (20) could be influenced by an altered thymic development and peripheral homeostasis through factors like CD28, IL-2, or TGF-ß (63, 64) as Tregs are IL-2 and CD28-dependent for their development, activation and function (64–66) and TGF-ß is necessary for maintenance in the periphery (67, 68). A stable Treg phenotype is also important for the immunosuppressive function. The loss of characteristics including the constant expression of the Treg marker FOXP3, leads to a reduction of the anti-inflammatory and immune regulatory function (69).

In patients with active CD, the level of Tregs in the lamina propria was found to be increased, indicating a resistance to Treg-mediated suppression or defective function (70, 71). A defective Treg function (**Figure 3C**) could result from insufficient expression of cell surface receptors, such as CTLA-4 or LAG3, which are important for the inhibition of dendritic cells. Another dysfunction in regulating immune responses could occur due to a failure of the production and secretion of granzyme A or B, as well as soluble factors, such as IL-10, IL-35, or TGF-ß (63, 72). The deficiency of IL-10 has been demonstrated to induce intestinal inflammation, and a Treg lacking IL-10 receptors has been observed to result in immune-mediated colitis (73).

Through the high level of pro-inflammatory immune cells at the site of intestinal inflammation, altered levels of cytokines such as, IL-1ß, IL-6, IL-12, and TNF-α are observed in the inflamed mucosa or lamina propria (74–77). The higher levels of such pro-inflammatory molecules have been shown to promote the proliferation of pro-inflammatory immune cells, such as effector T cells and, consequently, enhance resistance to Treg suppression (78, 79). It is hypothesized that the presence of a cytokine-rich milieu on the other side could potentially enable a limited function of Tregs (72) and could decrease a stable FOXP3 expression (69). Therefore, the efficacy of Tregs to suppress Teffs is reduced (**Figure 3D**).

In the context of therapeutic development, it is essential to expand Tregs and enhance the stability of their phenotype. Furthermore, it is necessary to support their suppressive function. Moreover, the specific guidance of improved Tregs to the inflammatory site may be necessary. This can be achieved by engineering of CARs that respond to proteins overexpressed at the site of inflammation. The engineered Treg specificity has the potential to result in more specific migration of anti-inflammatory cells to the effector site and thus increasing the local anti-inflammatory milieu. This could potentially increase their therapeutic success.

## 3 Chimeric antigen receptors for antigen-specificity

**Figure 4:**
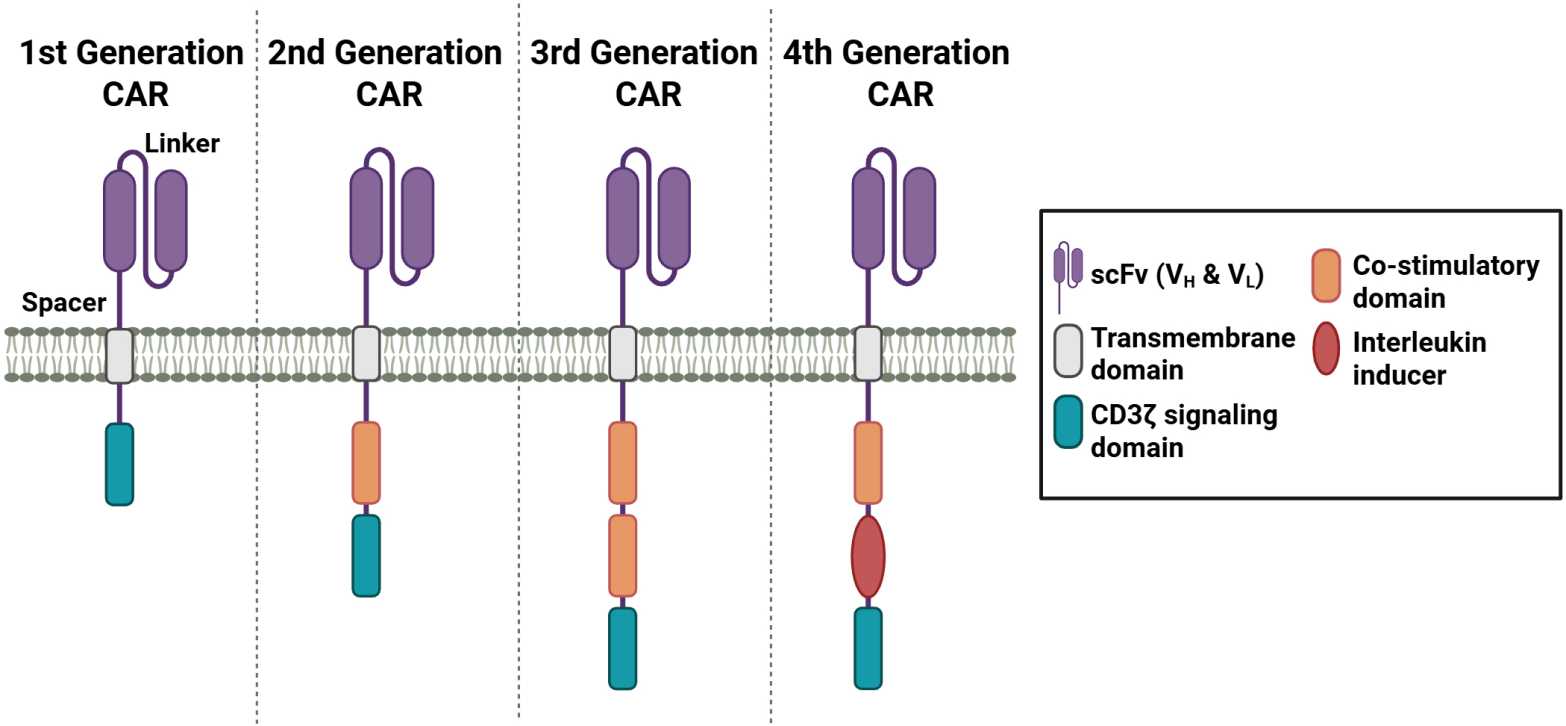
Different generations of CAR receptors. CAR receptors contain a scFv domain, which is connected through a spacer sequence to the transmembrane domain. Depending on the generation, one or more co-stimulatory domains and a CD3 signaling domain are incorporated. The fourth generation of CAR receptors contains an interleukin inducer domain. (Created in https://BioRender.com).

One novel tool to generate antigen-specificity is the use of chimeric antigen receptors (CARs). CARs are synthetically designed to bind to specific target antigens. The advent of CAR T cells can be traced back to 1989, when Zelig Eshar and Gideon Gross first developed engineered T cells (80, 81). Since then, there has been significant progress in the development of CAR constructs, with the emergence of more than four generations of these engineered receptors (**Figure 4**). The utilization of CARs confers numerous benefits, including the capacity to overcome HLA restrictions and enhance antigen specificity (18, 82, 83).

The target antigen is bound through a single-chain variable fragment (scFv) recognition domain originating from an antibody, which allows the specificity of the CAR construct. The scFv domain contains a light (V_L_) and heavy (V_H_) variable fragment, which are connected through a linker sequence. The order of the variable fragments, as well as the length of the linker sequences, has been demonstrated to influence the affinity and the stability of the receptor (84–86). The spacer domain contains sequences from CD28 or CD8α or from CH2 or CH3 domains of IgG1, 2, or 4 (87, 88). The length of the spacer domain has been shown to influence the distance between the effector and target cell (84). To anchor the CAR construct, a hydrophobic alpha helix transmembrane (TM) domain is connected to the spacer domain. The TM sequence of the first generation of CARs is derived from the CD3 TM domain (89). In the subsequent generations of CAR constructs, transmembrane sequences from CD4, CD8α, or CD28 were employed (87).

The first generation of CARs only consists of the T cell receptor (TCR) signaling chain CD3ζ, which functions to mediate T cell activation (90). However, the stimulation proved insufficient to achieve T cell expansion and elicit substantial therapeutic responses. In the subsequent second generation, an intracellular co-stimulatory domain, such as CD28 or 4-1BB, was incorporated. Studies have demonstrated that engineered CAR constructs with 4-1BB exhibited advantages in the persistence, while CARs with CD28 co-stimulatory domain revealed more rapid T cell activation (91–93). Third generation of CAR constructs were designed to enhance the T cell activating signal. Thus, two co-stimulatory domains from CD28, 4-1BB, OX40, CD27, or ICOS were combined (94). The incorporation of CD28 and an intracellular domain from the TNFR family into third-generation CAR constructs has been shown to facilitate the simultaneous activation of disparate signaling pathways (95, 96). The fourth CAR generation is based on the second-generation CAR and includes one co-stimulatory domain and an interleukin inducer domain, which enables the inducible release of IL-12 and IL-18 upon CAR activation (97, 98). This release of IL-12 or IL-18 creates a pro-inflammatory environment by recruiting and activating other immune cells, which in turn generate an immune response through further cytokine secretion and the promotion of exocytosis processes via granzyme or perforin. The fourth generation of CAR T cells are also referred to in the literature as TRUCKS, an acronym for “T cells redirected for antigen-unrestricted cytokine-initiated killing” (97–100). Subsequent exploration of additional CAR constructs was conducted in the context of next-generation CARs. One notable example involves the incorporation of a truncated IL-2 receptor β-chain domain, accompanied by a STAT3 binding site, within a next-generation CAR design (99).

The initial application of engineered CAR T cells was in the context of cancer therapies. Consequently, the initial documented success in these therapeutic interventions pertains to the administration of anti-CD19-CAR T cells in patients with advanced leukemia (101). Since then, the possibilities of CAR-T cell therapy have also been transferred to other diseases including allergic asthma (102), diabetes (103, 104), and IBD (105–107). Pre-clinical studies encompass an expanding array of CAR Treg models. A notable example is the development of CAR Tregs specific to β-amyloid for the treatment of Alzheimer’s disease. The efficacy of these cells has been assessed using murine brain lysates in preliminary studies (108). CAR Treg models for diabetes, including insulin B chain (InsB)-specific CAR Tregs, were developed and demonstrated suppressive effects in an adoptive transfer model of T cell-induced autoimmune diabetes in immunodeficient NOD mice, as reported by Spanier et al. (104). A number of HLA-A2-specific CAR Treg models have been engineered for utilization in transplant research, with an objective to avert graft-versus-host disease (GvHD) (109, 110). A number of Phase 1 studies of CAR Treg models are currently underway, including a study of CD6-CAR Tregs for the treatment of patients with chronic GvHD after an allogeneic hematopoietic cell transplantation (HCT) (111). The objective of this Phase 1 study is to ascertain the safety and tolerability of the intervention, as well as to assess its feasibility. In addition, the CAR Treg activity and persistence, as well as the alterations in immune biomarkers, will be monitored (111). A further clinical Phase 1/2 study includes HLA-A2 CAR Tregs (TX200-TR101) for a treatment of living doner kidney transplant recipients (112). The study’s objective is to assess the safety and tolerability of the treatment regimen, in addition to the range of doses that are administered. Additionally, immunosuppression and CAR Treg localization through renal transplant biopsies will be observed (112). A study of a similar nature utilizes HLA-A2 CAR Tregs (QEL-001) in order to prevent liver transplant rejection, as well in a clinical Phase 1/2 study (113). A further example is a clinical Phase 1 study with CAR Tregs specific for citrullinated proteins (Cit-P) for the treatment of refractory rheumatoid arthritis (114). As demonstrated in a series of pre-clinical and early clinical trials, CAR Tregs exhibit therapeutic potential for further applications in immune-related diseases.

## 4 Antigen-specific CAR Tregs in IBD therapies

As previously stated, Tregs possess the capacity to modulate the activities of immune cells, thereby regulating the inflammatory process in an anti-inflammatory manner. To this end, various models of genetically engineered CAR Tregs have been developed for the treatment of IBD using three different antigens upregulated in patients with IBD, including CEA-specific, Flagellin-specific CAR Tregs and IL23R-CAR Tregs.

### 4.1 CEA-specific CAR Tregs

The circulating carcinoembryonic antigen (CEA) has been studied as a tumor marker for colorectal cancer (115). However, in patients with inflammatory bowel disease, elevated CEA levels have also been observed (105). The levels were associated with the type, duration, and severity of the disease, as well as with the age and surgical status of the patients (115). Thus, Blat et al. generated a CAR targeting CEA (105).

In the study, the analysis of CEA-specific CAR Tregs was undertaken to ascertain their therapeutic potential in patients suffering from colitis (105). A stable phenotype of these cells, characterized by the expression of FOXP3 in the engineered CAR Tregs, was observed. They demonstrated the characteristic enhancement in IL-2 and IFN-γ secretion by anti-CEA CAR T cell activation and an enhancement of the IL-10 level following anti-CEA CAR Treg activation upon antigen exposure. Subsequent suppression assays showed an improved suppression of CD4+ effector T cells through anti-CEA CAR Tregs when stimulated by CEA antigen. In this study, a decrease in IL-2 levels and a suppression of Teff proliferation were observed. Furthermore, the study noted that i.p. injected CEA-specific CAR Tregs accumulated in the colon of diseased CEABAC-10 mice (105). CEABAC is a human “bacterial artificial chromosome” that contains part of the CEA family gene cluster, which consists of the CEACAM5, CEACAM3, CEACAM6, and CEACAM7 genes (116). The name CEABAC-10 refers to the number of copies of the CEABAC transgene in the mouse model’s genome (116). The expression of CEA is predominantly observed within the gastrointestinal tract (117, 118). Membrane anchorage of CEA is attributed to two distinct groups: glycophosphatidylinositol (GPI)-anchored members, including CEACAM5, CEACAM6, and CEACAM7, and transmembrane members, such as CEACAM3 (116). In a transfer colitis mouse model, which is achieved by injection of anti-CEA CAR CD4+ Teffs, suppressive effects and inhibitory effects on abdominal accumulation of the anti-CAR CD4+ Teffs were achieved by treatment with CEA-specific CAR Tregs. The CAR Tregs showed also in the AOM-DSS model colitis-suppressive effects (105).

### 4.2 Flagellin-specific CAR Tregs

An aberrant composition of gut microbiota has been associated with the onset of inflammatory bowel diseases. IBD lesions are frequently observed in the terminal ileum and colon, which are known to harbor the highest concentrations of bacteria (119). The identification of dominant bacterial antigens in IBD has highlighted flagellin as a pivotal antigen, particularly in Crohn’s disease. The major subunit of the bacterial flagella is flagellin (119, 120). The flagellum enables the bacteria to move into the intestinal mucus layer (121). The antigen flagellin has been identified as a ligand for Toll-like receptor (TLR) 5 on the surface of innate and adaptive immune cells, including Tregs, and epithelial cells (106). Through interaction of flagellin with the TLR5 receptor, the production and secretion of cytokines and co-stimulatory molecules were initiated in immune cells, such as T cells, monocytes or NK cells (122). This results in an inflammation reaction. It is notable that the colon mucosa is remarkably susceptible to colon flagellin, while an intact colonic mucosa remains unresponsive to luminal flagellin (123). As previously mentioned, TLR5 is also expressed in Tregs, which are known for their immune regulatory and anti-inflammatory functions (124). This characteristic renders flagellin a potential target for Treg therapy.

Boardman et al. (106) engineered a second-generation CAR construct targeting flagellins, which derived from *Escherichia coli* H18 (FliC). Following transduction with the CAR receptor, the engineered Tregs exhibited a stable phenotype, characterized by the persistent expression of FOXP3 and Helios. The activation of the developed FliC-CARs was achieved in the presence of the target antigen, flagellin. This was observed by an upregulation of CD69, PD1, and GARP, as well as by increased secretion of IL-10. The immunosuppressive reactions were documented by the inhibition of the proliferation of CD4+ and CD8+ responder T cells. In order to investigate the potential of the FliC-specific CARs to guide the engineered cells to damaged intestinal tissues, FliC-CAR T cells were injected intravenously in immunodeficient NSG mice. An accumulation of the FliC-CAR T cells in the colon was observed (106). The study by Boardman et al. (106) demonstrates the potential of specific genetically engineered CAR constructs on regulatory T cells for IBD therapies, based on identified overexpressed or highly available target antigens at the inflammation site.

### 4.3 IL23R-CAR Tregs

Genome-wide studies have revealed an association of Crohn’s disease with the IL-23 signaling pathway through the IL-23 receptor (IL23R) (125), thereby emphasizing an essential role of IL-23 in the pathogenesis and manifestation of IBD (126). The IL23R expression has been found to be elevated in the intestinal tracts of patients with Crohn’s disease (107). Furthermore, the presence of specific T cell subsets at the intestinal inflammatory site is influenced by secreted IL-23 cytokines. In patients with ulcerative colitis (UC) and Crohn’s disease (CD), an increased production of IL-23 by myeloid cells or neutrophils has been observed (126, 127). Additionally, IL23R signaling in T cells has been shown to inhibit FOXP3 expression, thereby resulting in the inhibition of differentiation of peripheral naive T cells to pTregs (128).

Cui et al. (107) generated specific CAR Tregs targeting IL23R for the treatment of CD disorders. It was observed that the IL23R-CAR expression did not modify FOXP3 expression in the Tregs. The study further demonstrated the capacity of these CAR Tregs to induce antigen-specific immunosuppression through the inhibition of CD4+ T cell proliferation. Subsequent analyses further confirmed the ability of engineered CAR Tregs to suppress DC function and maturation. The study emphasized that the selection of CAR constructs with the lowest tonic signal in the absence of stimulation was crucial to prevent Treg exhaustion and the loss of suppression function. This finding underscores the crucial role of CAR design in modulating the function of CAR Tregs. In this regard, IL23R-CAR Tregs have emerged as a promising therapeutic candidate for the treatment of CD, exhibiting characteristics such as antigen-specific immunosuppression and *in vivo* migration into IL23R-expressing tissue (107).

These three CAR Treg cell products show the immense potential of CAR Treg therapy for patients in IBD (**Table 1**). Although all three antigen-targets show a disease-specific increase in expression level, the risk of off-site effects remains a problem. For example, CEA is also highly expressed in colon cancer (115), limiting the therapeutic window for this CAR-construct. Similarly, off-target effects can be caused by the CAR Treg with flagellin specificity, the major subunit of bacterial flagella, due to the high bacterial concentration in the gut (119). The IL-23 receptor is expressed on the surface of T cells (128), which could influence immune homeostasis at sites without inflammation.

**Table 1:** CAR Treg model summary.

CAR Treg	Antigen	Characteristics antigen	Off-target risks/disadvantages	Therapeutic potential
CEA-specific CAR Treg	Carcino-embryonic (CEA) antigen	CEA: - known as soluble tumor marker of colorectal cancer (115) - increased level in ulcerative colitis (105) - glycophosphatidylinositol-anchored protein on intestinal epithelial cells (129, 130)	- CEA level different according to type, duration & severity of IBD (105) - potentially severe side effects in patients with non-diagnosed malignancy expressing CEA	- healthy tissue low levels of CEA (131) - CEA rise in IBD leading to a good disease-specific targeting opportunity
Flagellin-specific CAR Treg	Flagellin	Flagellin: - major subunit of bacterial flagella (120) - increased level in Crohn´s disease (120)	- high concentration of bacteria in gut (119) can lead to off-site effects	- intact colonic mucosa remains unresponsive to luminal flagellin (123) - Flagellin is a ligand of TLR5 receptor, which is expressed on Tregs and can additionally activate Tregs (124) - TLR5 signaling enhances FOXP3 expression in Tregs (124)
IL23R-CAR Treg	IL-23 receptor (IL23R)	IL-23 receptor: - enhanced IL-23 signaling through IL23R in Crohn´s disease (125) and T cell-mediated colitis (126) - IL23R expressed on T cell surface (128)	- can also influence immune homeostasis in healthy environments	- good migration to pro-inflammatory environments, which is mediated by high levels of infiltrating T cells (107)

The different CAR Treg models are displayed, along with their off-target effects and therapeutic potential.

## 5 Current challenges and future possibilities

Current therapeutic interventions for IBD, encompassing anti-cytokine treatments and immune cell trafficking/signaling blockers, exhibit limited efficacy (16). Consequently, the use of Tregs has emerged as a promising therapeutic strategy. As antigen-specific Tregs are more potent, genetically engineering CARs for antigen-specificity is a promising therapeutic approach. CARs can be tailored to suit specific requirements. The incorporation of a 4-1BB co-stimulatory domain has been shown to enhance persistence, while a CD28 co-stimulatory domain facilitates more expeditious signaling activation. The modular system can be utilized to develop new and optimized CAR T cells with longer persistence, improved stability, and faster signaling activation or coupling of other mechanisms. Genome-wide studies of IBD have enabled the identification of antigens associated with the disorder, which could be used for additional antigen-specific CAR constructs (125, 132, 133). Therefore, the potential use of IBD-dysregulated epithelial barrier proteins as antigens is possible. Examples include mucus layer protein Muc1 (Mucin-1) and desmosomal proteins such as Dsg3 (Desmoglein-3) (133).

For the regulatory function of Treg cells, the stable expression of FOXP3 as a master transcription factor is essential (134). It has been shown that repetitive *in vitro* stimulation of human Tregs with anti-CD3 and anti CD28 coated beads leads to loss of FOXP3 expression (135–137). Thus, the application of a Treg therapy in a disease of chronic inflammation could lead to a loss of FOXP3. The stabilization of the expression of this transcription factor could be addressed through the targeting of epigenetic modifiers, such as DNA methyltransferases, histone deacetylases, histone demethylases or methyltransferases, including EZH2 (138, 139). The expression patterns subsequent to Treg activation are influenced by EZH2 methyltransferase (139). Additionally, it has been demonstrated to directly regulate FOXP3 expression (139, 140). A further study observed enhanced expansion and an improvement in FOXP3 expression in response to a combination of IL-2, rapamycin, histone deacetylase, and DNA methyltransferase inhibitors (141). In other studies, an improved and stabilized FOXP3 expression was reported through the addition of vitamin C (142). Treg destabilization upon repeated TCR-stimulation might be rare (143–145), but the effect of repeated CAR stimulation on Tregs remains to be fully examined. Thus, it is worth further investigation considering the supraphysiological signaling through a CAR (146, 147).

In order for CAR Treg treatment to be applied in the clinic, there are a number of further key issues that need to be addressed before its clinical translation. Aside from showing the efficacy of CAR Treg treatment, the safety of this cell therapy will become paramount. The implementation of safety mechanisms, such as suicide programs, which have been developed for CAR effector T cell therapies, can be transferred to CAR Treg therapies. The incorporation of suicide cassettes has been demonstrated to serve the purpose of preventing dysfunctions or depleting the transferred engineered cells (138). Suicide genes that are currently under development include truncated epidermal growth factor receptor (EGFRt) and RQR8 (148). EGFRt is co-expressed with the CAR receptor, and the transferred cells can be eliminated with the application of an EGFRt-directed antibody, cetuximab (149). RQR8 combines parts from CD34 and CD20 antigens. The administration of CD20-directed antibodies, such as rituximab, has been demonstrated to enhance the susceptibility of target cells to lysis (148). Additionally, cells expressing RQR8 could be sorted by CD34 (148). The engineered CaspaCIDe, a safety switch technology, possesses the capability to, through the addition of rimiducid as the trigger, induce the dimerization of the CID domains in cases of toxicity. The subsequent initiation of the apoptosis pathway is catalyzed by iCasp9 (Caspase9) (150).

In other studies, the administration of dasatinib, a tyrosine kinase inhibitor, has been demonstrated to exert effects that block T cell function, with these effects being reversible upon removal (151). The efficacy of this effect may also extend to CAR Tregs.

In addition to efficacy and safety, ensuring the universal accessibility of CAR Treg therapy is imperative. A notable constraint pertains to the financial burden associated with CAR Treg therapy, in addition to the necessity for specialized equipment (17, 152). The provision of CAR Tregs as personalized products is a process that necessitates a substantial duration of production out of patient blood, accompanied by challenges related to the quality of the expanded cell product. Furthermore, IBD patients exhibit reduced Treg cell numbers, which consequently leads to challenges in isolating a sufficient number of cells and formulating a personalized therapy (152, 153). An enhancement in accessibility might be achievable through the standardization of the product and the process as well as the implementation of cryopreserved batches (154, 155). Off-the-shelf products are consistently available. However, they also reveal issues related to cell number, quality, and the time required to generate them. Additionally, HLA (human leukocyte antigen) compatibility is a critical factor in this process. In order to prevent alloreactivity, the generation of banks with different common HLA haplotypes is a potential solution (155). Alternatively, gene editing methods, such as the CRISPR-Cas9 system, can be utilized to eliminate T cell receptors (TCRs). One method involves the direct encoding of CAR DNA in the TRAC locus (156). Other methods involve the rendering of cells HLA-deficient (154, 157).

**Figure 5:**
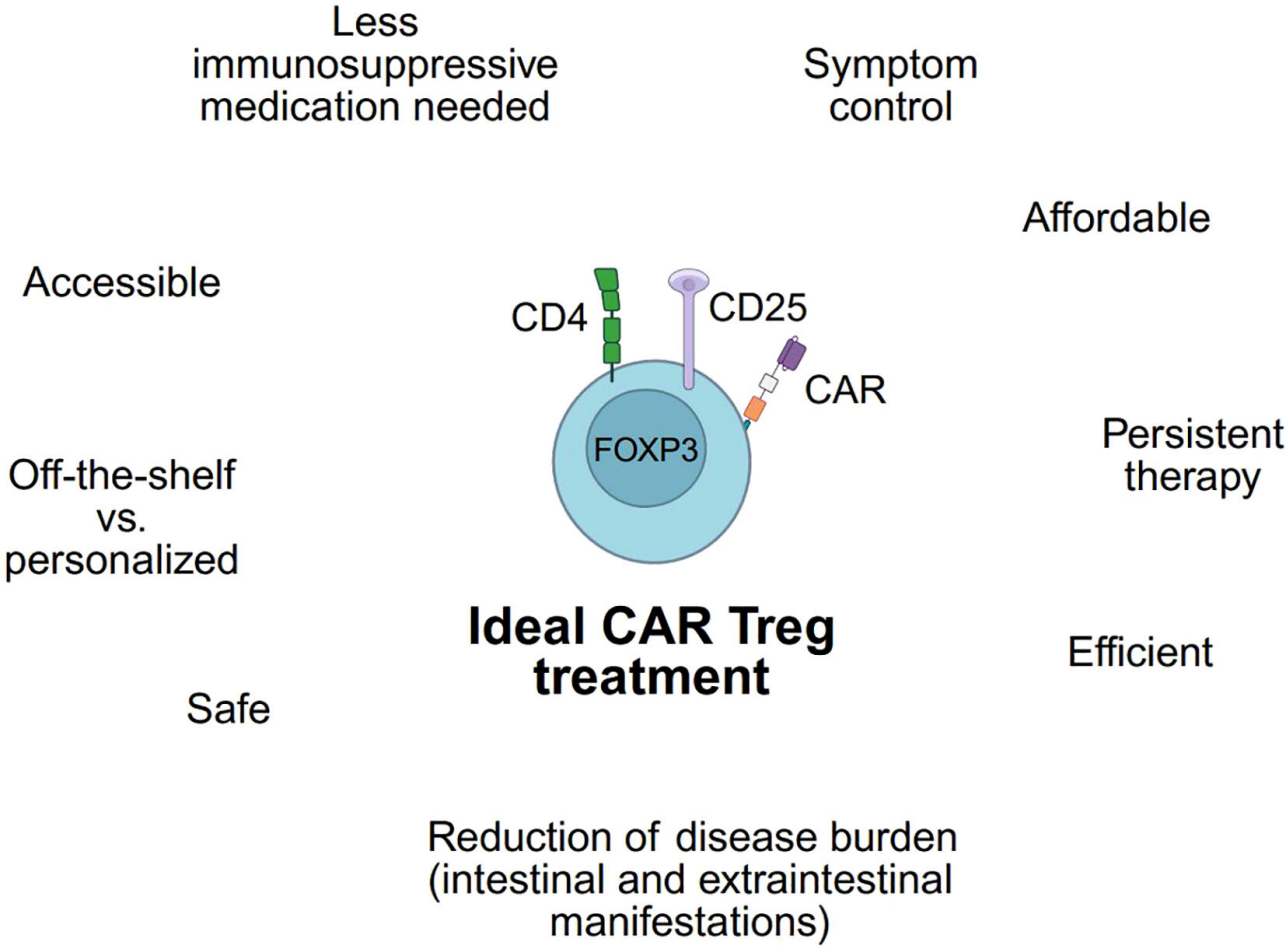
Requirements of an ideal CAR Treg. Utilizing a CAR Treg product to treat IBD patients depends on fulfilling numerous criteria. These requirements are presented for an ideal CAR Treg. (Created in https://BioRender.com).

Many preclinical and clinical trials will have to be conducted before the ideal CAR Treg therapy for patients with IBD can become standard-of-care. This ideal CAR Treg product not only should be efficient, persistent, and safe but lead to a significant reduction in symptom and disease burden while remaining accessible and affordable (**Figure 5**). Whether an off-the-shelf product or a personalized product with autologous Treg will be a more feasible approach needs to be addressed in future research.

## 6 Conclusion

Current IBD therapies are associated with limited success and severe side effects. As Tregs exert anti-inflammatory effects and the ability to suppress pro-inflammatory immune cells, the use of Tregs for the treatment of IBD is a promising approach. Using genetically engineered CAR constructs to guide Tregs to the site of inflammation can significantly enhance their function and therapeutic potential. These CARs have been developed and improved in function and efficacy in several CAR generations in recent years. The design of an appropriate CAR construct with respect to the antigen-specific target and the use in IBD opens new therapeutic opportunities. In preclinical studies, the anti-CEA, anti-flagellin, and anti-IL23R-CAR Tregs have been shown to be promising first developments for the treatment of IBD. Nevertheless, safety concerns must be addressed for clinical translation. The development of suicide programs, such as the incorporation of suicide genes (e.g., EGFRt and RQR8) or the utilization of CaspaCIDe technology in engineered Tregs to enhance safety, has emerged as a pivotal area of focus in contemporary studies, with the objective of facilitating clinical translation. Moreover, the issue of accessibility must be addressed by considering universal useable CAR Tregs that are devoid of allorejection reactions. In addition, the development of strategies to prevent CAR Treg exhaustion and to enhance a stable phenotype and suppressive function could be addressed through the addition of factors against epigenetic modifiers. Such modifiers include DNA methyltransferases, histone deacetylases, histone demethylases, and methyltransferases.

After considering the current limitations, the use of targeted, engineered Tregs to treat inflammation bowel diseases appears to be a promising therapeutic approach to overcome the limitation of current therapies.
